# Compound Radix Sophorae Flavescentis exerts antitumor effects by inhibiting the proliferation and inducing the apoptosis of esophageal carcinoma TE-8 cells

**DOI:** 10.3892/ol.2015.3607

**Published:** 2015-08-13

**Authors:** XIAOYU YANG, WEIMEI CAI, QINGHUI YANG, ZHIHONG LU, JINSONG LI, JIAN YU

**Affiliations:** 1 Department of Pathology, Xinxiang Medical University, Xinxiang, Henan, P.R. China; 2 The First Affiliated Hospital of Xinxiang Medical University, Xinxiang, Henan, P.R. China

**Keywords:** compound Radix Sophorae Flavescentis, esophageal carcinoma, apoptosis

## Abstract

The aim of this study was to examine the effects of compound Radix Sophorae Flavescentis on the proliferation of esophageal carcinoma TE-8 cells and to elucidate the mechanisms involved. For this purpose, we incubated TE-8 cells in medium containing various concentrations (0, 0.0125, 0.025, 0.05, 0.1, 0.2, 0.4 and 0.8 mg/ml) of the compound Radix Sophorae Flavescentis injection and its effects on the proliferation of TE-8 cells were examined by 3-(4,5-dimethylthiazol-2-yl)-2,5-diphenyltetrazolium bromide (MTT) assay. In addition, we observed the morphological changes and measured the expression levels of apoptosis-related genes (caspase-3, Bcl-2 and Bax) in the cells treated with different doses of the compound (low-dose group, 0.05 mg/ml; medium-dose group, 0.2 mg/ml; and high-dose group, 0.8 ng/ml) by reverse transcription-quantitative PCR (RT-qPCR). The apoptotic index of the cancer cells treated with different doses of the compound was determined by TUNEL assay. Our results revealed that compared with the control group (untreated cells), the proliferation of the cancer cells treated with the compound was significantly inhibited (P≤0.05); the inhibition of the proliferation of the cancer cells occured in a dose-dependent manner. Compared with the control group, the apoptotic rate of the cells in the low-dose, medium-dose and high-dose groups increased significantly (P<0.05) in a dose-dependent manner. In addition, compared with the control group, the mRNA expression of caspase-3 and Bax increased significantly in the cells treated with the compound. However, the mRNA expression of Bcl-2 markedly decreased (P<0.05). With the gradual increase in the drug concentration, the mRNA expression levels of caspase-3, Bcl-2 and Bax in the cancer cells were altered in a dose-dependent manner. In conclusion, our data demonstrate that compound Radix Sophorae Flavescentis injection significantly enhances the expression of pro-apoptotic genes in esophageal carcinoma TE-8 cells by increasing apoptosis and inhibiting cell proliferation. Thus, this study provides a theoretical basis for the clinical treatment of esophageal carcinoma.

## Introduction

Esophageal carcinoma is one of the most common types of malignant tumors in China (1). Due to the lack of specificity of symptoms, early-stage esophageal carcinoma is difficult to detect and some patients have already entered into the advanced stages of the disease at the time of diagnosis. Chemoradiotherapy is currently the main treatment strategy (2–5) for patients with advanced-stage esophageal carcinoma. However, the adverse effects associated with chemoradiotherapy undermine the effectiveness of treatment and the quality of life of patients.

In recent years, the continuous development and study of traditional Chinese medicine herbal treatments has obtained fruitful results. The main components of compound Radix Sophorae Flavescentis injection are Radix Sophorae Flavescentis and Heterosmilacis Japonicae extract, which can clear away heat, promote diuresis, cool blood, expel miasma, remove stasis and relieve pain (6). It has been demonstrated that CKI suppresses tumor cell growth by inducing apoptosis (7) and inhibits the migration, invasion and adhesion capacity of tumor cells by downregulating the protein expression of CD44v6 (8). Few studies to date have focused on the efficacy of Kushen alkaloids using animal models and performing clinical trials before 1992, when Kushen alkaloids were first approved. Studies have proven that the compound Radix Sophorae Flavescentis may be used to improve the efficacy of chemoradiotherapy and may also improve the quality of life of patients (6,9). Clinical studies have reported that Kushen alkaloids are efficacious in the treatment of various types of solid tumors (including tumors of the lung, liver and gastrointestinal tract). The treatment responses were comparable to, or better than, those of chemotherapy drug-treated patients (10). Kushen alkaloids demonstrate a good safety profile in cancer patients, such as reduced toxicity in bone marrow when used in combination with chemotherapeutic agents (11). Long-term survival data for Kushen alkaloids-treated cancer patients remain to be demonstrated with well-controlled clinical studies and large patient cohorts. However, studies on the mechanisms of action of this compound are limited. Thus, in this study, we analyzed the anti-neoplastic effects of compound Radix Sophorae Flavescentis, as well as its mechanisms of action in *in vitro* (using esophageal carcinoma TE-8 cells), with the aim of providing a theoretical basis for the clinical treatment of esophageal carcinoma.

## Materials and methods

### 

#### Experimental materials

##### Cell culture

Human esophageal squamous cell carcinoma TE-8 cells were provided by the Laboratory of Reverse Transcriptase Molecular Biology, Xinxiang Medical College, Xinxiang, China. The cells were cultured in RPMI-1640 medium supplemented with 100 U/ml penicillin, 100 µg/ml streptomycin and 10% (V/V) fetal bovine serum (FBS) in an incubator at 37°C with 5% CO_2_. The medium was changed every 24 h and the cells were passaged once in every 48 h.

##### Primary reagents

The compound Radix Sophorae Flavescentis was provided by Shanxi Zhendong Pharmaceutical Co., Ltd., Changzhi, China; 3-(4,5-dimethylthiazol-2-yl)-2,5-diphenyltetrazolium bromide (MTT) was obtained from Jingke Biotechnology Co., Ltd., Beijing, China; the apoptosis assay kit was from the Beyotime Institute of Biotechnology, Haimen, China; terminal deoxynucleotidyl transferase-mediated dUTP nick-end labeling (TUNEL) reagent was purchased from Roche Diagnostics, Mannheim, Germany; the reverse transcription kit was obtained from Nanjing Vazyme Biotech Co., Ltd. (Nanjing, China); SYBR-Green universal qPCR Master Mix was from Roche Diagnostics; the real-time PCR system was purchased from Bio-Rad (Hercules, CA, USA); and the inverted fluorescence microscope was obtained from Leica Microsystems (Mannheim, Germany).

#### Treatment groups

Esophageal carcinoma TE-8 cells were inoculated in 96-well plates at a density of 5×10^4^ cells/ml, and kept in an incubator at 37°C with 5% CO_2_ for overnight culture. The following day, the adherent cells (60–70% adherence) were placed in RPMI-1640 medium supplemented with 0.2% FBS, and the supernatant was extracted after 24 h. The compound Radix Sophorae Flavescentis injection was then added to the medium at various concentrations (final concentrations: 0, 0.0125, 0.025, 0.05, 0.1, 0.2, 0.4 and 0.8 mg/ml) with a total of 8 gradients, followed by culture for 24 h. There were 4 different treatment groups: the control group (untreated cells), the low-dose group (0.05 mg/ml of the compound), the medium-dose group (0.2 mg/ml of the compound) and the high-dose group (0.8 ng/ml) of the compound. Cell proliferation was then examined by MTT assay. The plate was removed, abd 20 µl MTT solution (5 mg/ml) were added to each well. The cells were then incubated at 37°C for 4 h. The culture medium was then removed, and the cells were washed with PBS, followed by the addition of DMSO. The mixture was then vortexted for 10 min, and the light absorption (OD value) in each well was measured at 492 nm and the readings were recorded. The experiment was repeated 3 times, and the mean measurements were calculated from sextuplicate samples. The cell growth inhibition rate (%) was calculated as 1 − (mean OD value in treatment group/OD value in control group) x100.

#### Reverse transcription-quantitative PCR (RT-qPCR) for the determination of the expression of apoptosis-related genes

The cells treated with various doses of the compound (low dose, medium dose and high dose (0.05, 0.2 and 0.8 mg/ml, respectively) were treated with 1 ml of TRIzol reagent (Beyotime Institute of Biotechnology) and placed in a 1.5 ml centrifuge tube. Subsequently, 200 µl chloroform was added, and the tube was shaken vigorously (for mixing) and then place on ice for 15 min to layer, followed by centrifugation at 12,000 rpm/min for 15 min. When the liquid appeared as the layer, the supernatant was placed in 500 µl isopropanol, on ice, after mixing until the RNA precipitated out and was then centrifuged at 12,000 rpm/min for 10 min. The precipitate was washed with 75% pre cooling anhydrous ethanol twice; it was then dissolve in double-distilled water without RNA enzyme. Following the detection of the concentration of sample, the RNA was reverse transcribed into cDNA using the reverse transcription kit. The primers used for PCR were as follows: caspase-3 forward, 5′-GGTATTGAGACAGACAGTGG-3 and reverse, 5′-CATG GGATCTGTTTCTTTGC-3; β-actin forward, 5′-GCGGGAA ATCGTGCGTGAC-3′ and reverse, CGTCATACTCCT GCTTGCTG-3′; Bax forward, 5′-TCCACCAAGAAGCT GAGCGAG-3′ and reverse, 5′-GTCCAGCCCATGATG GTTCT-3′; Bcl-2 forward, 5′-TTCTTTGAGTTCGGTGG GGTC-3′ and reverse, 5′-TGCATATTTGTTTGGGGCAGG-3′. The reaction mixture was prepared using the following reaction system: 2X SYBR-Green universal 10 µl qPCR Master Mix, 1 µ upstream/downstream primer (10 µmmolxl^−1^), 1 µl cDNA and 20 µl double-distilled water. The following reaction conditions were used for PCR: initial denaturation, 95°C, 30 sec; degeneration, 95°C, 3 sec; annealing and extension, 60°C, 30 sec; building of solubility curve. Finally, the data were analyzed using LightCycler software.

#### Calculation of apoptotic index TUNEL assay

The TE-8 cells were digested and kept in monoplast suspension, and inoculated in a culture dish containing a glass slide with at 1×10^5^ cells/ml; the groups and treatment conditions were the same as those described above. The medium was removed after 48 h, the glass slide was removed and TUNEL assay was carried out according to the instructions provided by the manufacturer. The slide was removed, washed with PBS for 3 times, fixed in 4% paraformaldehyde for 40 min, and washed with PBS for 3×3 min. The slide was blocked at room temperature for 10 min, washed with PBS for 3×3 min, placed at 4°C for 3 min, washed with cold PBS for 2×5 min, followed by the addition of labeling mixture. It was then incubated in a dark and wet environment at 37°C for 60 min, washed with PBS for 3×5 min, followed by the addition of 50 µl transformation solution. It was then incubated in a dark and wet environment at 37°C for 30 min, and washed with PBS for 3×5 min again. DAB was then added and the slide was observed under a microscope. The slide was restained with haematoxylin, dehydrated, transparentized and mounted on a microscope for observation. TUNEL-positive cells were indicated by a brown nucleus. The cells were then observed under a microscope at at 5–10 fields of vision on a high-power lens (x400 magnification) so that each field of vision included 100–200 cells. The total number of cells was approximately 500–1,000 and the proportion of cell apoptosis was calculated as the apoptotic rate.

#### Statistical analysis

All the data was analyzed using statistical software SPSS 17.0, and the data are expressed as the means ± SD. Comparisons among groups were made by one-way analysis of variance (ANOVA), and pairwise comparisons were made using the LSD method. The survival curve was estimated using the Kaplan-Meier method and the log-rank test. A value of P<0.05 was considered to indicate a statistically significant difference.

## Results

### 

#### Effect of compound Radix Sophorae Flavescentis injection on the proliferation of esophageal carcinoma TE-8 cells

**Figure 1.**
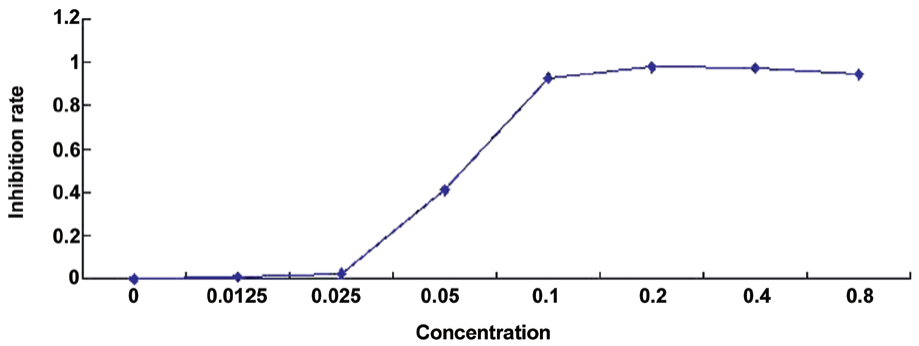
Effect of compound Radix Sophorae Flavescentis on the proliferation of esophageal carcinoma TE-8 cells. Inhibitory effect of various concentrations of Radix Sophorae Flavescentis on the proliferation of TE-8 cells.

As shown in Fig. 1, compared with untreated control group, cell proliferation was significantly inhibited in the cells treated with the compound in a dose-dependent manner (P<0.05).

#### Effect of compound Radix Sophorae Flavescentis injection on the expression of apoptosis-related genes in esophageal carcinoma TE-8 cells

**Figure 2.**
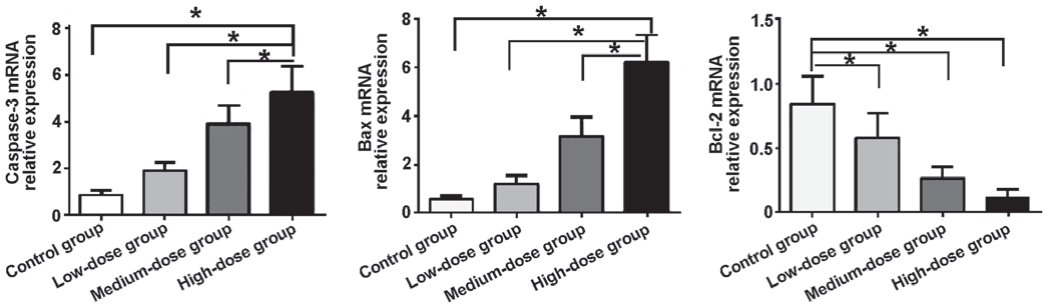
Effect of compound Radix Sophorae Flavescentis on mRNA expression of apoptosis-related genes (caspase-3, Bcl-2 and Bax) in esophageal carcinoma TE-8 cells. The cells were treated with various doses of the compound (low-dose group, 0.05 mg/ml; medium-dose group, 0.2 mg/ml; and high-dose group, 0.8 ng/ml) and the mRNA expression levels were measured by RT-qPCR. *P<0.05.

After the cancer cells were treated with various concentrations of the compound Radix Sophorae Flavescentis, the mRNA expression levels of the apoptosis-related genes, caspase-3, Bcl-2 and Bax, were measured by RT-qPCR and the results are shown in Fig. 2. Compared with the control group, the mRNA expression levels of caspase-3 and Bax significantly increased in the cells in the low-dose, medium-dose and high-dose groups, whereas the mRNA expression level of Bcl-2 markedly decreased (P<0.05). The increase in the mRNA expression of caspase-3 and Bax in the different treatment groups was as follows (in the order of lowest to highest): low-dose group<medium-dose group<high-dose group. The decrease in the mRNA expression of Bcl-2 in the different treatment groups was as follows (in the order of highest to lowest): low-dose group>medium-dose group>high-dose group.

#### Effect of compound Radix Sophorae Flavescentis injection on the apoptotic index in esophageal carcinoma TE-8 cells

**Figure 3.**
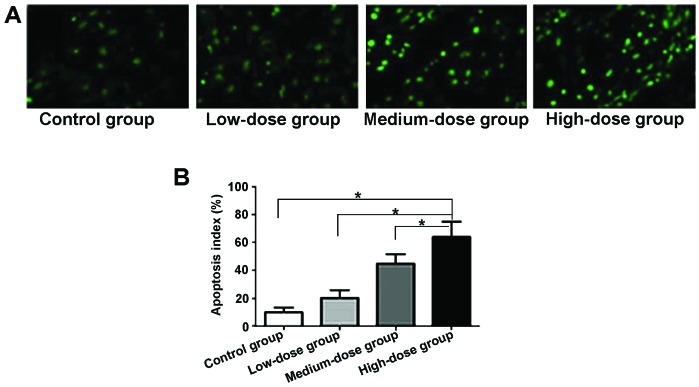
Effect of compound Radix Sophorae Flavescentis on the apoptotic index in TE-8 cells. (A and B) The cells were treated with various doses of the compound (low-dose group, 0.05 mg/ml; medium-dose group, 0.2 mg/ml; and high-dose group, 0.8 ng/ml) and the apoptotic index was calculated by TUNEL assay. *P<0.05.

The apoptotic index in the cancer cells was calculated using TUNEL asay and the results are presented in Fig. 3. The number of apoptotic the cells in the control group was low; however, following treatment with increasing doses of the compound, cell apoptosis gradually increased. The quantitative analysis of the results of cell apoptosis is shown in Fig. 3B. Compared with the control group, the apoptotic index of the cancer cells in the low-dose, medium-dose and high-dose groups increased significantly and the difference was statistically significant (P<0.05). The apoptotic index in the different gropus ranged as follows (in the order of highest to lowest): high-dose group>medium-dose group>low-dose group.

## Discussion

Esophageal carcinoma is one of the most common gastrointestinal malignancies within the category of ‘hiccough’ and ‘phrenic’ in traditional Chinese medicine and its pathogenesis is ‘qi, phlegm, blood stasis and heat’ (12). Thus, the treatment of esophageal carcinoma should aim to clear heat, transform phlegm and soften hardness. The main components of the compound Radix Sophorae Flavescentis injection are Radix Sophorae Flavescentis and Heterosmilacis Japonicae extract, which can clear heat, promote diuresis, cool blood, expel miasma, remove stasis and relieve pain (13).

It has been demonstrated that the compound Radix Sophorae Flavescentis injection exerts antitumor effects and can alleviaate pain, bleeding, fatigue and other discomforts (14). It has also been reported that (15) that matrine can kill malignant tumor cells and promotes cancer cell apoptosis. In this study, we investigated the antitumor effects of the compound Radix Sophorae Flavescentis injection by treating esophageal carcinoma TE-8 cells with different doses of the compound and found that the compound significantly inhibited the proliferation of the cancer cells in a dose-dependent manner. Thus, the compound Radix Sophorae Flavescentis injection inhibits the proliferation of esophageal carcinoma cells.

In this study, we also measured the mRNA expression of apoptosis-related genes in esophageal carcinoma TE-8 cells and the results revealed that the mRNA expression of caspase-3 and Bax was significantly higher in the cells treated with a high dose of the compound (0.8 mg/ml) compared with the control group, whereas the mRNA expression of Bcl-2 markedly decreased in the cells treated with the compound compared with control group. Caspase-3 is a common molecule of the cell apoptosis pathway, which plays an important role in the process mediating apoptosis (16). An increase in its expression promotes apoptosis. In our study, treatment with the compound Radix Sophorae Flavescentis injection induced an increase in the expression of caspase-3, resulting in the apoptosis of the esophageal carcinoma cells. The balance of Bcl-2 and Bax also plays an important role in the process of apoptosis in cancer cells, with the increase in Bcl-2 expression indicating the inhibition of apoptosis, and the decrease in Bax expression maintaining the proliferation of cancer cells (17–19). In our study, following treatment of the cancer cells with the compound Radix Sophorae Flavescentis injection, Bcl-2 expression markedly decreased and Bax expression significantly increased, indicating an increase in apoptosis. The induction of apoptosis may be one of the mechanisms through which the compound Radix Sophorae Flavescentis injection inhibits the growth of tumors. Cancer cell apoptosis caused by compound Radix Sophorae Flavescentis injection was further verified by TUNEL assay.

In conclusion, our study demonstrated that compound Radix Sophorae Flavescentis injection significantly inhibited the proliferation of esophageal carcinoma TE-8 cells and induced apoptosis. Thus, compound Radix Sophorae Flavescentis injection may prove to be a valuable and effective therapeutic agent for the treatment of esophageal carcinoma.
